# Understanding recurrence in *Mycobacterium avium* complex pulmonary disease: genotypic strategies to support clinical decision-making

**DOI:** 10.1128/jcm.01086-25

**Published:** 2025-11-05

**Authors:** Minh Phuong Trinh, Sung Jae Shin, Min-Kyoung Shin

**Affiliations:** 1 Department of Microbiology and Convergence Medical Science, College of Medicine, Gyeongsang National University 26720, Jinju, Republic of Korea; 2 Department of Microbiology, Institute for Immunology and Immunological Diseases, Brain Korea 21 PLUS Project for Medical Science, Yonsei University College of Medicine 37991, Seoul, Republic of Korea; University of Western Australia, Perth, Western Australia, Australia

**Keywords:** clinical decision-making, genotyping, relapse and reinfection, recurrence, *Mycobacterium avium* complex (MAC)

## Abstract

Pulmonary disease caused by *Mycobacterium avium* complex (MAC-PD) is a chronic, recurrent disease, and its high recurrence rate after treatment makes clinical management difficult. Distinguishing whether recurrence is due to persistence of existing strains or reinfection with new strains is essential for establishing treatment strategies, preventing overuse of antimicrobials, and establishing infection control measures. According to reports, 54%–74% of MAC-PD recurrence is due to reinfection, which may be mainly related to environmental reservoirs such as household water supply. In this review, we present various clinical scenarios in which MAC-PD recurrence may occur and examine genotyping techniques as a strategy to distinguish and respond to them. From traditional methods such as IS*1245*-based restriction fragment length polymorphism, pulsed-field gel electrophoresis, and *hsp65* and *rpoB* gene sequencing to high-resolution analysis techniques such as multilocus sequence testing and whole-genome sequencing, the latest molecular typing methods are comprehensively summarized. Integrating these genotype data into clinical settings, standardizing single-nucleotide polymorphism-based interpretation thresholds, and promoting the establishment of a global MAC strain database will make a substantial contribution to more accurately distinguishing the recurrence mechanisms of MAC-PD and establishing personalized treatment strategies.

**IMPORTANCE:**

The global burden of nontuberculous mycobacterial pulmonary disease (PD) is increasing, with *Mycobacterium avium* (MAC)-PD being the most prevalent and clinically challenging form. Its low treatment success rates, high frequency of recurrence, and persistent environmental exposure complicate both diagnosis and management. A critical clinical issue is determining whether recurrence represents true relapse, due to persistence of the original strain, or reinfection with a new strain, as this guides treatment and prevents overtreatment. Genotypic strategies capable of resolving strain-level differences can improve diagnostic accuracy, prevent misclassification, and ultimately support more informed treatment decisions. Therefore, integrating genotyping data into clinical workflows, standardizing single-nucleotide polymorphism thresholds, and establishing a global MAC strain database will not only support personalized treatment but also enhance the broader public health response to this disease.

## INTRODUCTION

Pulmonary disease caused by nontuberculous mycobacteria (NTM-PD) has become increasingly worldwide, driven in part by global population aging (1). Among NTM species, *Mycobacterium avium* complex (MAC), mainly consisting of *M. avium* and *M. intracellulare*, is the most frequently isolated group in human infections, accounting for 27%–85% of NTM-PD cases, with especially high prevalence reported in East Asia and North America (1–4). In clinical practice, MAC treatment generally includes macrolide-based combination therapies given for a minimum of 12 months after culture conversion. However, only about 60% of patients attain a lasting microbiological cure, whereas roughly 40% either do not respond to treatment or experience a relapse (5–7). Additionally, in individuals who attain initial clearance, reinfection, frequently caused by new strains obtained from the environment, continues to be a major issue, with reported recurrence rates between 32% and 48% (8, 9). This phenomenon of recurrence is a major clinical and public health concern in MAC-PD. Recurrence involves various mechanisms, including persistence of the original strain and introduction of new strains from the environment (10). While these mechanisms may appear clinically similar, they have distinct prognostic and therapeutic implications. Therefore, accurate strain identification is essential for appropriate assessment of clinical outcomes and optimization of treatment decisions (10).

Molecular typing techniques such as restriction fragment length polymorphism (RFLP), pulsed-field gel electrophoresis (PFGE), and multilocus sequence typing (MLST) have greatly improved the capacity to distinguish among MAC strains, each providing different levels of discriminatory resolution over the past two decades (11–13). In addition to treatment outcomes, these technologies can provide information on environmental sources, transmission patterns, and outbreak control (12, 13). When applied in clinical and epidemiological settings, genotypic data can meaningfully inform decision-making and enable more targeted interventions (14). This review provides a comprehensive overview of genotyping approaches used to achieve strain-level differentiation in MAC-PD. We attempt to provide the resolution, diagnostic utility, and clinical applicability of each strategy in differentiating types according to the recurrence mechanism. Finally, we aim to establish a foundational perspective for implementing more individualized, genotype-informed strategies in the clinical management of MAC-PD.

## KEY DEFINITIONS AND INTERPRETIVE CRITERIA FOR MAC RECURRENCE

MAC-PD recurrence after termination of treatment is a prevalent and clinically significant issue (5, 15, 16). However, the term “recurrence” encompasses multiple underlying mechanisms that are distinct in both etiology and clinical consequence. Accurate terminology is essential for guiding treatment decisions, designing studies, and interpreting microbiologic and epidemiologic data.

Recurrence broadly refers to the reappearance of symptoms and culture positivity after a period of clinical improvement and microbiological conversion (17). Within this definition, two main mechanisms can be delineated: relapse and reinfection. Relapse is defined as disease recurrence caused by the same strain that was responsible for the original infection (18–23). Reinfection, in contrast, refers to a new infection event caused by a genetically distinct strain of MAC, usually acquired from environmental reservoirs (21). Although the two mechanisms may present similar clinical features, distinguishing them is clinically important, as species-level identification is insufficient, and strain-level genotyping, such as WGS or MLST, is necessary to assess clonal relatedness and determine whether relapse is due to persistence or *de novo* acquisition (12, 21).

## CLINICAL SCENARIOS OF MAC RECURRENCE

**Fig 1:**
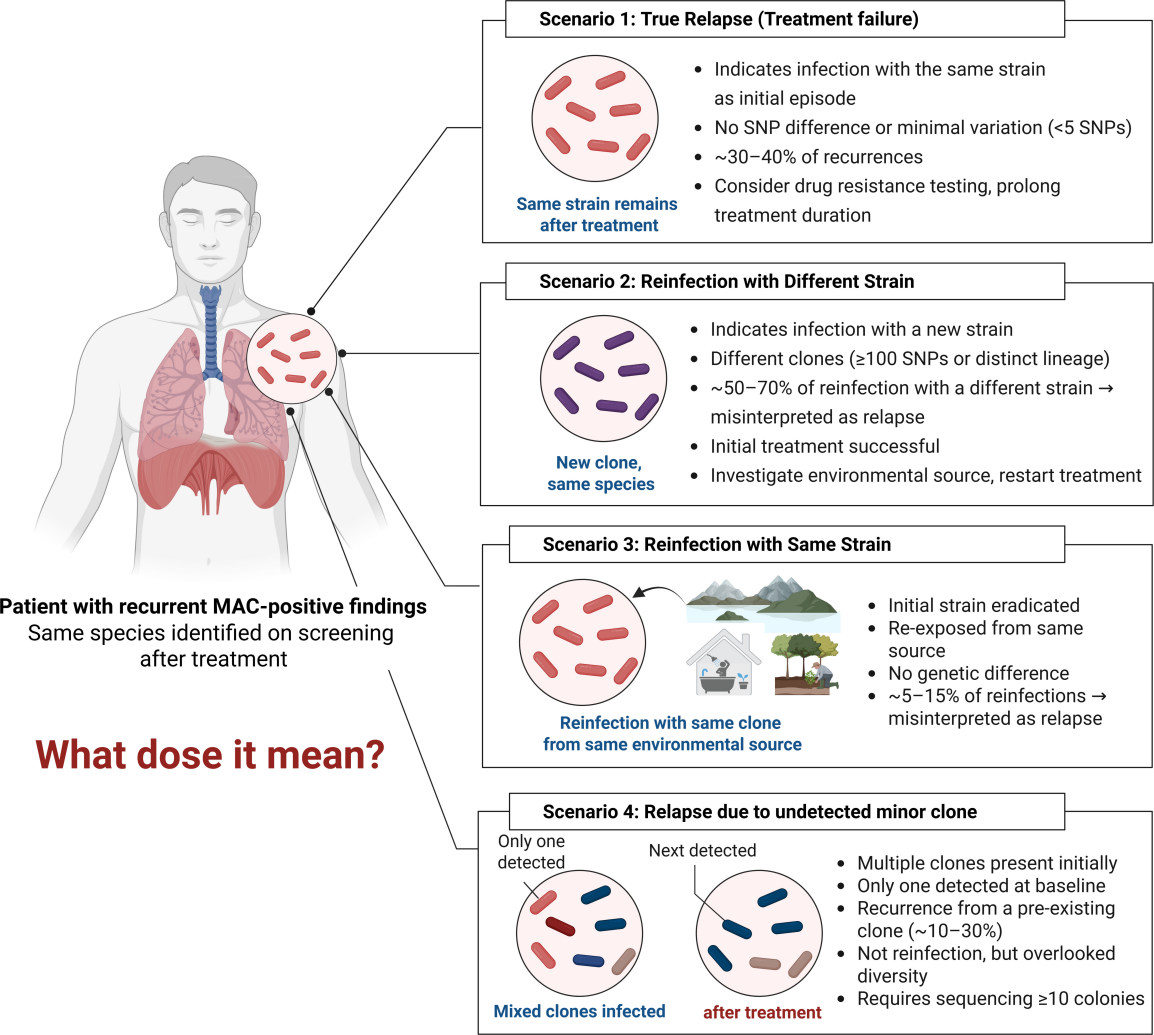
Interpretation scenarios of recurrent MAC-PD in clinical settings. This schematic outlines four potential explanations for the recurrence of MAC-PD after therapy: (1) True relapse (2), reinfection by a genetically distinct strain (3), reinfection with the same strain, and (4) recurrence due to an undetected minor clone. These categories are defined by treatment outcome, environmental re-exposure, and differences in genomic similarity between isolates. MAC-PD, *Mycobacterium avium complex* pulmonary disease; SNP, single-nucleotide polymorphism.

In clinical practice, recurrence of MAC pulmonary disease is far from rare, even after apparently successful treatment. Yet, behind the generic term “recurrence” lies a spectrum of biologically distinct events that demand closer scrutiny. Although the traditional dichotomy of relapse versus reinfection provides a useful framework, recent evidence suggests that the clinical reality is more complex. Recurrence may arise from one of at least four distinct scenarios, each with its own origin, diagnostic considerations, and therapeutic implications (Fig. 1).

The first and most widely recognized is true relapse. In this scenario, the patient experiences a resurgence of disease caused by the same bacterial strain as the initial infection. This typically reflects incomplete bacterial clearance during initial therapy, often due to the presence of persistent organisms in protective niches such as biofilms or within host macrophages (24, 25). Relapse is frequently associated with antimicrobial resistance or subtherapeutic drug levels and requires prompt reassessment of drug susceptibility and possible regimen escalation.

The second scenario involves reinfection with a genetically distinct strain. In this situation, the original strain has been eradicated, but the patient acquires a new MAC strain from the environment. Such cases are especially prevalent in endemic regions where environmental sources like tap water, showerheads, soil, or aerosols act as reservoirs of MAC (23). In contrast to relapse, this situation does not signify a failure in treatment; instead, it demonstrates persistent susceptibility to host or environmental influences. In such instances, treatment choices can range from starting a new therapy regimen to implementing environmental interventions or maintaining vigilant clinical observation.

Third, a more diagnostically ambiguous situation is reinfection with the same clone. In this situation, the original infection has been cleared, and the patient is re-exposed to an identical genotype from the same environmental reservoir. Because the recurrent isolate cannot be distinguished from the initial strain by genotyping, this scenario is often misclassified as a relapse. Without supporting environmental or epidemiologic data, even high-resolution genomic tools may be insufficient to differentiate these two origins (26). Therefore, even if genotyping results alone indicate a relapse, it may actually be a reinfection, and in this case, the clinical approach should be different, such as maintaining the current treatment rather than initiating a new treatment rather than starting a new treatment, or choosing close observation without active intervention, unlike scenario 1. Importantly, this scenario highlights the need for epidemiological investigations into the environmental ubiquity of MACs and the resulting exposure sources.

Finally, polyclonal infections, where multiple genetically diverse strains coexist within a patient from the outset, can complicate recurrence classification. Single-colony testing, a standard practice, may miss minority strains, leading to misinterpretation of a relapse as a reinfection if a previously undetected clone becomes dominant during recurrence (27–30). The study by Iwamoto et al. demonstrated the wide genetic diversity of *M. avium* strains across humans, animals, and environmental reservoirs, as well as the possibility of genetically distinct *M. avium* clones to coexist in the same host or environment using VNTR-based molecular typing (31). This finding highlighted an important limitation of colony-based assays that rely on single or small numbers of isolates and emphasizes the imperative for multiple colony- or population-level genotyping strategies (31). To address this, guidelines recommend analyzing 3–5 colonies per strain and 5–10 isolates per outbreak to ensure robust strain comparisons and reduce selection bias (32). This approach enhances the accuracy of distinguishing relapse from reinfection in complex cases.

## GENOTYPING METHODS: APPROACHES TO RESOLVE CLINICAL UNCERTAINTY

Strain-level genotyping extends beyond its role as an epidemiological tool by providing evidence to distinguish relapse from reinfection in MAC-PD and to support clinical and microbiological interpretation. In this section, we review the principal genotyping techniques used in MAC management, outlining their strengths, limitations, and relevance to clinical decision-making. Comparative features of each method are summarized in Table 1, and representative clinical applications of individual genotyping methods are provided in Table 2.

**TABLE 1:** Advantages and disadvantages of individual genotyping methods for NTM and considerations for clinical application

Methods	Advantages	Disadvantages	Clinical setting (relapse vs. reinfection)
RFLP (IS*1245*, IS*1311*)	- Direct sample analysis from DNA - Moderate discriminatory power for MAC	- Time-consuming, complicated - Poor reproducibility - Not well standardized	- Still used in some reference labs for MAC strain identification - Useful when high-resolution typing is not required
PFGE	- Good discrimination at the strain level - Higher resolution than RFLP - Good for outbreak analysis and strain tracking	- Technically demanding - Time-consuming - Less suitable for high-throughput analysis	- Valuable for differentiating relapse vs. reinfection in *M. abscessus* and MAC - Useful in epidemiological studies
Single-gene sequencing (16S rRNA)	- Simple, fast - Universal target for bacterial identification - Well-characterized and widely available	- Cannot distinguish between closely related species (e.g., in MAC) - Low resolution at the strain/subspecies level	- Useful for initial species identification
Single gene sequencing (*hsp65*)	- Better species-level resolution - Simple and low-cost - Commonly used in NTM species identification - Applicable to low-biomass samples	- Limited discrimination between strains - May miss minor variants or mixed infections	- Useful in resource-limited settings - Useful for initial species identification - Suitable for differentiating closely related species
Single gene sequencing (*rpoB*)	- Good differentiation within the *M. abscessus* complex group - Provides both species identification and resistance information (e.g., rifampin)	- Still lacks whole-genome context - Not all resistance mutations lie in canonical regions	- Dual-purpose marker for species typing and resistance detection - Helpful in relapse cases with suspected resistance
rep-PCR	- Relatively fast - High discriminatory power - Suitable for clinical labs	- Limited standardization and portability - Banding pattern analysis is subjective	- Useful in distinguishing relapse vs. reinfection in MAC - Best for within-institution comparisons
MIRU-VNTR	- Simple to perform, fast, and labor-saving and need only a small amount of DNA - Stable, and the evolution rate is slightly slower than RFLP - Good application in disease surveillance - High reproducibility - Digital, comparable across labs - Suitable for large-scale studies	- May lack resolution in very clonal populations - Requires established loci panels	- Limited use in NTM due to a lack of a suitable marker system - Ideal for longitudinal and outbreak surveillance - Well-suited to MAC and *M. abscessus* typing
Digital VNTR	- Long-read sequencing, PCR-free - 100% concordance with conventional VNTR - High precision and reproducibility in quantifying VNTR copy numbers - Generates standardized digital outputs for cross-laboratory comparison - Enables strain-level differentiation with ≥1 repeat change indicating a different strain	- Requires next-generation sequencing infrastructure - Potential cost and technical expertise needed - Limited validation in diverse NTM populations	- Effective for distinguishing relapse (stable dVNTR profiles) from reinfection (≥1 repeat change at any locus, indicating a different strain) - Suitable for molecular surveillance and routine clinical settings where WGS is less accessible
MLST	- Can be compared between labs - Sequence-based and portable - Excellent for population studies	- Time-consuming - Lower resolution than WGS - Requires a standard database - May miss microevolution during chronic infection	- Can distinguish reinfection if the sequence type is different - Well-suited to global comparisons via PubMLST
cgMLST	- Subspecies-level resolution - 99% accuracy for species ID - Can be integrated with resistance prediction (rrl, rrs, erm [33]) - High portability with mlstverse	- Less discriminatory than SNP-based WGS	- Useful for distinguishing species/subspecies shifts - Combined with dVNTR for strain-level surveillance
Whole-Genome Sequencing	- Highest resolution - Accurate discrimination of relapse/reinfection - Detection of drug resistance and strain evolution - Detects SNPs, resistance genes, mixed infections - Enables phylogenetic analysis	- High cost - Requires bioinformatics - Interpretation standards are still evolving - Lack of standardized interpretation thresholds (e.g., SNP cutoffs for relapse vs. reinfection may vary by species and study)	- Gold standard for distinguishing relapse vs. reinfection - Enables personalized treatment decisions and source tracking
tNGS (Targeted NGS)	- Rapid and cost-effective sequencing of large DNA samples (days vs. weeks for culture) - Culture-independent; works on paucibacillary samples - Focuses on specific genomic regions for in-depth analysis - High specificity in detecting NTM species with mNGS - Direct analysis of clinical samples without culture - Revolutionizes genomics by enriching target regions with probes	- Limited to predefined targets (misses novel variants) - Requires prior knowledge of resistance loci - Moderate cost; bioinformatics needed - Longer turnaround time than mNGS due to targeted analysis	- Useful for initial drug resistance screening in relapse cases (e.g., accurate MTB/NTM diagnosis) - Differentiates reinfection via targeted SNP analysis in known hotspots; complements WGS for resource-limited NTM surveillance
mNGS (Metagenomic NGS)	- Broad, unbiased detection of all nucleic acids in a sample - High sensitivity for NTM in mixed infections (>80%) - Detects non-culturable/rare strains and multiple NTM species (e.g., MAC, *M. intracellulare*, *M. abscessus*) - Shorter turnaround time than tNGS; high throughput for outbreaks - The area under the curve (AUC) up to 0.916 in bronchoalveolar lavage fluid samples	- High cost and turnaround time - Host DNA interference reduces specificity - Data overload requires advanced bioinformatics - Less specific than tNGS for targeted NTM detection	- Ideal for complex reinfection scenarios (e.g., polyclonal NTM in extrapulmonary cases) - Distinguishes relapse via strain stability in longitudinal samples; useful for environmental NTM tracking and rapid diagnosis in bronchoalveolar lavage fluid/sputum
MGIT-seq	- 99.1% accuracy for NTM species ID; 84.5% for subspecies - Predicts macrolide/AMK resistance with > 97% specificity - Reduces TAT (hours vs. days); integrates with liquid culture - No need for subculturing	- Dependent on MGIT culture positivity first - Limited to sequenced isolates (not direct from sputum) - Requires MinION/ONT setup and NGS infrastructure	- Supports relapse detection via resistance tracking (e.g., 19.4% macrolide resistance in MAC) - Identifies reinfection shifts in NTM-PD cohorts; practical for clinical decision-making in treatment-refractory cases
MinION (Nanopore Sequencing)	- Portable, real-time sequencing with long reads for VNTR/SNP resolution - Low cost per run; field-deployable - Enables dVNTR/cgMLST integration for strain shifts - Specialized software/databases for precise NTM ID	- Higher error rate (~5–15% raw; improvable with polishing) - Needs computational resources for assembly - Variable yield in low-biomass NTM samples	- Facilitates on-site relapse vs. reinfection genotyping (e.g., ≤10 SNPs for relapse) - Tracks environmental reinfection in NTM-PD; bridges lab-to-field gaps
MALDI-TOF	- Rapid (minutes), low-cost species ID (>95% accuracy for common NTM) - Minimal sample prep; high-throughput - Identifies based on unique spectral fingerprints - Distinguishes *Mycobacterium tuberculosis* complex from NTM; low consumable costs	- Limited to species level (poor strain resolution; challenges with closely related NTMs) - Database gaps for rare NTM - Requires extraction for tough samples like mycobacteria	- Quick initial screening for relapse (stable spectra) - Limited for reinfection (needs follow-up genotyping); useful in low-resource settings for NTM with TB differentiation
CRISPR	- High specificity for spacer-based strain discrimination - Rapid, low-cost detection of variants; multiplexed for outbreaks - CRISPR-Cas12a for NTM detection; components include ss reporters, Cas effector, gRNA - Potential for genome editing tool-based assays	- Limited to CRISPR loci (misses genome-wide changes) - Design complexity for custom spacers; emerging validation needed for NTM diversity - Extensive sample processing and equipment costs	- Targets CRISPR spacers for relapse (identical arrays) and reinfection (spacer loss/gain) - Promising for phylogenetic tracking in clonal NTM populations
AI/ML	- Optimizes MLST schemes (e.g., reduces loci by 10× while retaining >90% accuracy) - Analyzes complex data (genomics, images) for SNP/VNTR prediction - Improves outbreak clustering from WGS; AUC up to 0.94 for NTM-LD detection - Models like SVM, random forests for histopathological analysis and marker selection	- Requires large training data sets and high-quality genomic data - Black-box interpretability issues - High computational demands; needs infrastructure for WGS/tNGS/mNGS integration	- Enhances relapse/reinfection calls via pattern recognition in cgMLST data (e.g., 90% accuracy in NTM ID) - Automates strain typing for surveillance; reduces bias in NTM epidemiological studies (e.g., differentiates NTM from TB with AUC 0.84)

**TABLE 2:** Clinical implementation of genotypic tools to differentiate relapse and reinfection in NTM pulmonary disease

Genotyping method	Species	Patients	Key finding	Implication	Clinical implication	Geographic region	References
PFGE	MAC	481	- Species differences affect prognosis - 26% relapses, 74% reinfections	NB form, higher recurrence risk	*M. intracellulare,* poorer outcomes	South Korea	Koh et al. (34)
PFGE PFGE + Genotyping	MAC MAC (NB form)	46	- 54% true relapse - 46% reinfection with a new strain	Relapse linked to clarithromycin; faster recurrence	Genotype correlates with resistance & timing	USA	Boyle et al. (21)
		481	- NB phenotype as an independent risk factor - 74% reinfection	Clinical phenotype + strain typing guide therapy	NB form may need tailored treatment and long-term follow-up	South Korea	Koh et al. (34); Lee et al. (9)
RFLP	*M. avium*	63	- Polyclonal infections confirmed - Multiple genotypes coexisted in a single host	Within-host heterogeneity is common	Highlights the importance of genotyping multiple colonies	Italy	Lari et al. (35)
RepPCR (in-house primers) + VNTR	*M. intracellulare*	25 / 101	- Novel rep-PCR using five species-specific primers produced seven distinct fingerprint patterns among clinical isolates - 95%–98% reproducibility - High correlation with VNTR (r = 0.814)	Strain-level genotyping of *M. intracellulare* is feasible without WGS	Aids in recurrence tracking and outbreak investigation when WGS is not available	South Korea	Shin et al. (36); Shin et al. (37)*
Single-gene sequencing (*rpoB*, 23s rRNA) + rep-PCR	MAC	72	- 27% relapse, 73% reinfection	Therapy adjustment vs environmental control	Adjust therapy for relapse; consider the environmental source for reinfection	South Korea	Jhun et al. (38)
MLST	MAC	15	- 54.5% reinfection, potential transmission - 45.5% persistent infections	Community transmission risk	Community-level risk; surveillance needed	Thailand	Boonjetsadaruhk et al. (39)
VNTR	*M. intracellulare*	74	- 50 genotypes identified - Discrimination index = 0.988 (16 loci)	High stability and resolution of VNTR	Reliable for epidemiological tracing and outbreak studies	Japan	Ichikawa et al. (40)
MIRU-VNTR + WGS	*M. abscessus*	Cystic fibrosis cohost	- Detected patient-to-patient transmission and genotype changes over time	Tracks transmission and detects reinfection/superinfection	Supports infection prevention and control policy and longitudinal genotyping in cystic fibrosis clinics	UK	Bryant et al. (10)
VNTR + WGS (environmental sampling)	MAC	37 households (21 with patients)	- 52.4% of patient isolates matched household plumbing genotypes - 85.7% of patient isolates had a genotype match with plumbing in the same community	The environment is a significant infection source	Water system surveillance and home plumbing disinfection are needed	USA	Lande L et al. (41)
cgMLST + dVNTR	MAC, *M. intracellulare*, *M. abscessus*	112 NTM-PD patients (prospective cohort)	- 25.9% experienced pathogen shifts over ~ 1.5 years - Species/subspecies change in 13 (11.6%) - Strain-level change in 16 (14.3%) - The Shift(−) group had higher macrolide resistance - A change of ≥ 1 repeat at any locus is considered sufficient to define a different VNTR type, thereby indicating a different strain	Dynamic strain turnover occurs even during treatment/observation - cgMLST + dVNTR feasible in routine clinical settings	Enables real-time monitoring of relapse vs. reinfection; Supports timely treatment adjustment and infection control	Japan	Hashimoto et al. (33)
WGS (SNP-based)^*a*^	*M. abscessus*	One cystic fibrosis patient	- Relapse confirmed by WGS (<3 SNP difference) - Strain and resistance profile identical to isolate one year earlier	WGS accurately distinguishes relapse from reinfection even in individual cases	WGS enables accurate differentiation even in single cases	USA	Chawla et al. (42)
WGS^*a*^	*M. abscessus*	31 adult cystic fibrosis patients	- Confirmation of human-to-human transmission through SNP analysis, prompting changes in hospital control policies	Strong evidence for nosocomial or indirect human transmission	Prompted revision of infection control policy in cystic fibrosis clinics	UK	Bryant et al. (10)
WGS^*a*^ tNGS (Targeted NGS	MAC	Various	- 73% of recurrent MAC infections due to genetically distinct strains	Reinfection is the predominant mechanism	Emphasizes environmental source control	South Korea, USA	Operario et al. (43)
	MAC	Clinical samples	- Accurate diagnosis of MTB/NTM - High specificity for species detection - Targets enriched regions for cost-effective sequencing	Targeted drug resistance screening enhances relapse detection	Guides therapy escalation in relapse; identifies reinfection for environmental intervention	Multi-regional	Buckwalter et al. (44) Murthy et al. (45)
mNGS (Metagenomic NGS)	NTM	27 patients	- Detected polyclonal NTM in 26% of reinfection cases - Stable strain in 80% of relapse samples	Reveals complex reinfection dynamics	Differentiates polyclonal reinfection from relapse; informs outbreak control	China	Wang et al. (46)
MGIT-seq	MAC	100 (prospective cohort)	- 84% concordance with WGS for subspecies ID - Macrolide and amikacin resistance were detected in 19.4% and 1.9% of MAC and *M. abscessus* isolates	Rapid ID supports relapse vs. reinfection tracking	Facilitates timely adjustment in treatment-refractory cases	Japan	Fukushima et al. (47)
MinION (Nanopore Sequencing)	NTM	Various (field-deployable)	- Real-time output for quick ID - Integrates with dVNTR/cgMLS	Portable genotyping for strain shifts	On-site differentiation of relapse vs. reinfection in endemic areas	Multi-regional	Murthy et al. (45)
MALDI-TOF	MAC	60 strains from clinical samples	- 95% of clinical samples (57/60) ≥ 1.8 score (high-confidence)	Rapid initial species confirmation	Screens for relapse; requires follow-up genotyping for reinfection	Europe	Rindi L et al. (48)
CRISPR-based typing	*Mycobacterium* spp.	Clinical samples	- High specificity -Detects/differentiates NTM via Cas12a	High specificity for strain tracking	Targets relapse with reinfection in clonal outbreaks	Asia	Murthy et al. (45)
AI/ML in genotyping	NTM (disease-causing)	Various (e.g., imaging/ genomics)	- 90% accuracy for NTM markers; AUC 0.94 for NTM-PD detection - AUC 0.84 for differentiating NTM from MTB	Automates strain classification	Enhances surveillance and reduces bias in relapse/reinfection calls	Global	Murthy et al. (45)

^*a*^ Primarily, data from MAC pulmonary disease are presented, with studies on *M. abscessus* included where methodologically relevant. Insights from *M. abscessus* using high-resolution WGS also demonstrate the critical importance of accurate strain discrimination. Reinfection, particularly in nodular bronchiectatic forms or in settings of ongoing environmental exposure, frequently contributes to recurrence. Therefore, combining classical methods (e.g., rep-PCR, PFGE) with molecular targets (e.g., 23S rRNA mutations) or environmental sampling enhances diagnostic precision.

### Early fingerprinting methods: RFLP and PFGE

RFLP and PFGE were among the first molecular genotyping methods used to differentiate relapse from reinfection in NTM infections, particularly MAC-PD. RFLP, often using IS*1245* probes for *M. avium*, generates distinct banding profiles to distinguish similar and divergent strains (35) (Table 1). PFGE offers higher resolution by employing rare-cutting restriction enzymes to fragment large genomic DNA segments, separated via pulsed electric fields to produce strain-specific patterns (34). These methods have been widely applied in clinical studies to assess MAC recurrence. Koh et al. used PFGE to analyze 481 MAC patients, finding that 55% of recurrences involved the same strain, with 26% classified as relapses and 74% as reinfections, highlighting the predominance of environmental reinfection (34) (Table 2). Similarly, Boyle et al. employed PFGE in 46 patients, revealing that 54% of relapses showed unchanged banding patterns, often with higher macrolide minimum inhibitory concentrations (MICs) and earlier recurrence (210 vs. 671 days), suggesting treatment-related resistance (21) (Table 2). RFLP analysis by Lari et al. confirmed polyclonal infections in 63 *M*. *avium* isolates, revealing within-host heterogeneity that could be mistaken for relapse or reinfection without genotyping (35) (Table 2). Furthermore, PFGE has been studied to provide practical differential criteria for clinical interpretation, with relapses appearing as identical or very similar banding patterns (0–2 band differences), whereas reinfections appear as distinct patterns (>3 band differences) (34). These findings underscore the diagnostic utility of PFGE and RFLP in early MAC-PD studies, laying the groundwork for higher-resolution methods like whole-genome sequencing.

### Repetitive sequence-based methods: rep-PCR, MIRU-VNTR

Repetitive sequence-based polymerase chain reaction (rep-PCR) and mycobacterial interspersed repetitive unit-variable number tandem repeat (MIRU-VNTR) analysis are two widely applied molecular genotyping techniques used to assess strain diversity and track the genetic relatedness of NTM. Rep-PCR targets conserved, non-coding repetitive DNA elements such as ERIC, REP, and BOX sequences dispersed throughout the bacterial genome (49, 50). To amplify intermittent areas of varying length, these components function as primer-binding sites, producing DNA fingerprinting profiles that are extremely repeatable and selective. This technique is rapid, economical, and especially useful for strain-level differentiation in environmental and clinical isolates (Table 1). Rep-PCR has been shown in numerous epidemiological studies to be able to differentiate between reinfection and relapse in recurrent MAC infections, with reinfection accounting for a significant portion of cases post-treatment (8). Studies by Koh et al. and Wallace et al.*,* for example, have demonstrated that reinfection with genetically diverse strains accounted for the majority (up to 74%) of recurrent MAC cases, highlighting the significance of environmental exposure in disease recurrence (8, 34) (Table 2). Furthermore, rep-PCR may also effectively discriminate MAC isolates from recurrent cases, as demonstrated by Shin et al. (36) (Table 2). This suggests that reinfection with novel strains, rather than real relapse, accounts for a significant portion of treatment failures. In addition, Jhun et al. applied rep-PCR to sequential isolates from patients with refractory MAC-PD and showed that strain replacement with newly acquired environmental isolates frequently occurred during ongoing therapy, underscoring the clinical value of rep-PCR for identifying reinfection events even in treatment-refractory populations (38). For clinical interpretation, rep-PCR distinguishes relapse from reinfection based on identical versus divergent banding profiles, with studies reporting 95–98% reproducibility for strain-level differentiation (36).

On the other hand, VNTR genotyping is a molecular method that uses primers that target sequences that flank the VNTR sections to measure band widths produced by PCR amplification and electrophoresis to identify genetic diversity (51). Since the length of the repeat units is fixed, the resulting band sizes can indicate the quantity of VNTR copies in a specific strain, and the data are ultimately represented as the number of repeats at each genetic locus (51). These numerical data sets are especially beneficial for comparative analyses both within and between research laboratories and geographical areas. An important application includes the VNTR analysis of mycobacterial interspersed repetitive units (MIRUs), which are scattered throughout the *M. tuberculosis* genome, mainly in internal genomic areas. In contrast to rep-PCR, MIRU-VNTR offers a standardized, digital output suitable for global databases and epidemiological monitoring (26). Ichikawa et al. demonstrated the stability of 16 VNTR loci in *M. intracellulare*, distinguishing 50 genotypes from 74 clinical samples with a high discrimination index (0.988) (40). In addition to clinical applications, MIRU-VNTR is also useful in epidemiological surveillance and disease outbreak investigation. Although originally demonstrated in *M. abscessus*, the study by Bryant et al. showed that MIRU-VNTR combined with WGS can track transmission among cystic fibrosis patients and detect genotype changes within the same patient over time—a sign of reinfection or superinfection (10, 13) (Table 2). Overall, repetitive sequence-based methods such as rep-PCR and MIRU-VNTR remain valuable because of their clinical rapidity and accessibility, but the complexity of MAC relapse may necessitate the use of high-resolution tools for clear interpretation.

Building on conventional VNTR, Hashimoto et al. Recently, a digital VNTR (dVNTR) approach was proposed that leverages next-generation sequencing data to quantify VNTR copy numbers with greater precision and reproducibility (33). In a prospective cohort of 112 patients with NTM-PD, dVNTR, in combination with core genome MLST (cgMLST), successfully identified pathogen shifts, including both species/subspecies replacements and strain turnover with the same species (33). Importantly, dVNTR generates standardized digital outputs that can be directly compared across laboratories, enhancing its applicability for molecular surveillance and for distinguishing relapse (stable dVNTR profiles matching the original strain) from reinfection (divergent profiles). In this framework, a change of ≥1 repeat at any locus is considered sufficient to define a different VNTR type, thereby indicating a different strain (Table 2). VNTR is derived from genome-wide sequencing data and complements single-nucleotide polymorphism (SNP)-based analysis by providing an additional layer of strain differentiation and improving reproducibility across studies. In this way, dVNTR enhances the clinical applicability of high-resolution genomics for routine surveillance and for distinguishing relapse from reinfection in MAC-PD.

### Target gene sequencing

#### Single-gene sequencing

Species identification and subspecies-level classification within the MAC group have benefited greatly from target gene sequencing techniques, such as those that concentrate on the 16S rRNA, *hsp65*, and *rpoB* genes (52, 53) (Table 1). Although 16S rRNA sequencing is still a valid method for differentiating between genus and species, its capacity to distinguish across strains is limited by the high sequence conservation among *M. avium*, *M. intracellulare*, and *M. chimaera* (9, 37). Conversely, the *hsp65* gene, featuring hypervariable areas, provides better resolution and has been widely utilized for species identification and subspecies-level differentiation (53). Direct sequencing of *hsp65* has been used in several studies, but the more commonly used approach in resource-constrained environments is PCR-restriction fragment analysis (PRA-*hsp65*), which, however, has reduced discriminatory power when used on genetically similar strains (37, 54, 55).

Additional value is added by the *rpoB* gene, which codes for the RNA polymerase β-subunit and facilitates phylogenetic classification and rifampin resistance discovery (56). Rep-PCR and *rpoB* sequencing have been successfully used in recent research to improve strain-level resolution, especially for *M. intracellulare* (57). This combination offers a workable balance between resolution and practicalities, and it has shown performance comparable to multilocus techniques. A study by Jhun et al., which investigated 72 patients with MAC-PD, provided an example of how these genotyping methods might be used practically (38). Using rep-PCR and sequencing of the 23S rRNA region, the study found that 73% of recurrent cases represented reinfections with different strains, whereas 27% were true relapses involving the same strain (38) (Table 2). These findings highlighted the significance of genotyping in distinguishing between reinfection and relapse, guiding decisions about therapeutic adjustment versus environmental management, and supporting individualized treatment plans for NTM lung disease (38). A study by Koh et al. investigated 481 MAC-PD patients, finding that 74% of recurrences were reinfections with different strains, guiding decisions about therapeutic adjustment versus environmental management (34).

#### Multi-gene sequencing: MLST

MLST is a high-resolution, sequence-based genotyping technique that creates allelic profiles called sequence types (STs) by analyzing internal sections of several housekeeping genes, usually five to eight, including *recA*, *gyrB*, *rpoB*, *sodA*, and *argH* (Table 1). These genes are chosen because of their gradual evolutionary rates, consistent functionality, and lower vulnerability to selective pressures, rendering them perfect for extended phylogenetic and epidemiological studies (32, 58, 59). Boonjetsadaruhk et al. conducted MLST analysis using seven genes (*fusA, secA, rpoB, hsp65, 16S rRNA, 23S r*RNA, and ITS), demonstrating higher discriminatory power than conventional 4–9 gene sets and highlighting its utility as a diagnostic and epidemiological tool (39). In MAC infection, MLST has been particularly useful in distinguishing relapse from reinfection. The principle is straightforward: identical STs between initial and recurrent isolates indicate relapse, whereas different STs suggest reinfection (39, 40). Ichikawa et al. conducted MLST analysis on 74 *M*. *intracellulare* isolates, demonstrating a high reinfection rate post-treatment, suggesting that many cases previously considered treatment failures may reflect reinfection (60). Similarly, Uchiya et al. found several STs in serial isolates from single patients, indicating repeated reinfection instead of persistence (61) (Table 2).

MLST has proven reproducible and comparable across centers, supported by curated resources such as PubMLST (13). Despite these advantages, MLST has limited resolution, particularly in identifying microevolution or clonal differentiation after long-term antibiotic therapy. To overcome the limited resolution of conventional MLST, cgMLST was developed as a genome-wide extension that analyzes hundreds to thousands of conserved core genes shared across strains (62, 63). cgMLST provides far greater discriminatory power by leveraging high-density allelic profiles, thereby enabling the detection of microevolutionary changes and clonal differentiation during long-term infection or antibiotic therapy (62, 63). In MAC studies, cgMLST has already been applied to patient cohorts and shown to effectively track subspecies differentiation and long-term strain dynamics, particularly when combined with complementary methods such as VNTR or dVNTR (33). cgMLST may bridge the gap between conventional MLST and WGS-based approaches, offering a scalable and standardized framework for multicenter epidemiological surveillance (Table 1).

### High-resolution genomics: whole-genome sequencing

WGS offers the highest resolution for distinguishing relapse from reinfection in MAC-PD by analyzing the entire DNA sequence, including coding and non-coding regions (64) (Table 1). Fine-scale comparison of strains is made possible by the use of bioinformatic methods to detect SNP), insertions/deletions (indels), structural variations, and mobile genetic elements. WGS enables precise differentiation of relapse from reinfection using SNP-based thresholds. Typically, relapses are characterized by ≤5–10 SNPs of divergence between initial and recurrent isolates, while reinfections show >50–100 SNPs or distinct phylogenetic clusters (13, 38, 65). Applying WGS to MAC infections, Operario et al. showed that reinfection with genetically diverse strains was responsible for 73% of recurrent cases post-treatment, highlighting the importance of environmental acquisition (43) (Table 2). In clinical practice, accurate genotypic classification is crucial. Misidentifying reinfection as relapse may lead to unnecessarily prolonged therapy and increased drug-related toxicity—especially in vulnerable populations such as the elderly or immunocompromised. Long-term macrolide-based regimens, commonly extending beyond 12 months, are associated with adverse effects such as ototoxicity (amikacin), hepatotoxicity (rifampin), and gastrointestinal intolerance (clarithromycin) (5, 66). On the other hand, overlooking a true relapse can delay treatment intensification and promote antimicrobial resistance (21). Boyle et al. reported a clarithromycin resistance rate of 80% in relapsed cases, compared to 33% in reinfections, highlighting the clinical value of early genotypic differentiation (21). In endemic areas, where outside MAC strains are common and frequently genetically identical to clinical isolates, this problem is more severe. Additionally, WGS identifies polyclonal infections missed by single-colony testing, improving diagnostic accuracy (67). These capabilities have established WGS as the standard for MAC-PD recurrence analysis and are expected to support personalized treatment and environmental source tracking (68).

## CLINICAL IMPLICATIONS AND FUTURE STRATEGIES

Based on the clinical and epidemiological studies of MAC-PD reviewed above, several actionable strategies can be proposed that could substantially contribute to more individualized treatment decisions and effective public health interventions in the management of this disease.

First, a comparative analysis of serial strains in patients with MAC-PD recurrence should be incorporated as part of the clinical treatment process. In particular, a parallel approach to genotyping is necessary to accurately distinguish between relapse and reinfection and to support treatment decisions accordingly. High-resolution methods such as WGS and MLST can capture long-term strain evolution, while approaches that combine VNTR with cgMLST or integrate MIRU-VNTR with WGS have been successfully applied to detect dynamic strain replacement events (10, 33). Such findings emphasize the importance of continuous molecular surveillance in clinical practice, as these shifts may directly influence therapeutic decisions (33). Additionally, beyond patient-level genomics, environmental epidemiology also highlights that broader ecological factors shape NTM disease dynamics. Meteorological conditions and natural disasters have been identified as significant predictors of NTM incidence across diverse climate zones, suggesting that climate change and ecosystem alterations may increasingly affect the epidemiology of MAC-PD (69). These findings suggest that climate change and ecosystem alterations may increasingly influence the epidemiology of MAC-PD, underscoring the need to integrate molecular surveillance with environmental and public health data when developing prevention strategies (69).

Second, when interpreting genotypic information derived through high-resolution molecular analysis, it is necessary to establish internationally consistent standards. For example, the criteria defining reinfection as a difference of 50–100 or more SNPs and relapse as a difference of 5 or fewer SNPs have been suggested based on several WGS-based studies (10, 42). Furthermore, the intermediate range of 5–50 SNPs represents a “gray zone” where differentiation may be ambiguous, possibly reflecting microevolution or reinfection by a closely related species in relapsed cases. Recent studies, such as Wetzstein et al., suggested integrating clinical and epidemiological data (e.g., patient exposure history) with genomic analysis to resolve such cases, emphasizing the need for standardized thresholds and multi-colony analysis to improve accuracy (67).

Third, both initial diagnosis and follow-up cultures should use multiple-colony analysis. Selecting a single colony for genotyping is a common technique currently, which may not be sufficient to identify polyclonal infections. MAC infections can be mixed from the start, and if a new clone appears during recurrence without multi-colony analysis (≥10 colonies per specimen), it could be mistakenly identified as microevolution or reinfection.

Fourth, an international central database is needed for MAC strains, given the global prevalence of MAC and its increasing incidence in various regions. This repository should integrate high-resolution genome sequences, antimicrobial resistance information, clinical characteristics, geographical distribution, and treatment outcomes and could be similar to the TB-Profiler (https://tbdr.lshtm.ac.uk/) and PubMLST platforms for *M. tuberculosis* (https://pubmlst.org/organisms/mycobacterium-tuberculosis-complex). These resources can be used to track the emergence patterns of strains and the transmission routes within hospitals or communities and can provide practical support for clinicians to compare and analyze patient cases and establish optimal management strategies.

Fifth, an integrated system is needed to reflect genotypic information in actual clinical decision-making. Development of decision support algorithms that integrate clinical, molecular, and microbiological data is required, and it is expected to cover treatment response prediction, timely treatment adjustment, and environmental assessment.

Sixth, emerging diagnostic and analytic technologies are reshaping the management of NTM infections, including MAC-PD. Murthy et al. suggest that combining artificial intelligence and machine learning (AI/ML) with genomic approaches such as targeted or metagenomic sequencing could improve species and subspecies identification, predict resistance profiles, and distinguish closely related strains (45). Although these methods remain in early development, they may provide rapid and data-driven interpretations of complex genomic data sets, thereby supporting more precise distinction between relapse and reinfection and ultimately informing individualized treatment strategies (45).

CRISPR-based approaches are also emerging as valuable additions for MAC-PD. CRISPR arrays, with their hypervariable spacer regions, act as molecular barcodes to distinguish closely related MAC strains, detect polyclonal infections, and trace environmental sources with high precision (70). Recent CRISPR-based diagnostic platforms, such as SHERLOCK and DETECTR, have further expanded the potential of this approach by enabling rapid, sensitive, and multiplexed nucleic acid detection—even in resource-limited settings (71). In addition, integrated strategies combining optical DNA mapping with CRISPR-Cas9-guided targeting of resistance genes have shown promise for culture-free, polymicrobial, and plasmid-specific typing directly from clinical material (72, 73). Integrating CRISPR into existing genotyping workflows has the potential to improve diagnostic precision and enhance individualized patient management.

Other innovative platforms are also gaining traction. Targeted next-generation sequencing (tNGS) focuses on specific genomic regions for cost-effective detection of drug resistance mutations (44, 74, 75); metagenomic NGS (mNGS) can unbiasedly identify NTM in complex clinical samples (46, 76, 77); MGIT-seq links liquid culture with sequencing to enable accurate subspecies typing and resistance prediction (47); portable MinION nanopore sequencing offers real-time genomic readouts that are useful for field surveillance (45, 78); and MALDI-TOF mass spectrometry ensures quick species identification with spectral fingerprints (48). These tools are expected to fill the gaps in existing methods and facilitate the development of personalized treatment strategies (45) (Tables 1 and 2).

All these suggestions may provide a strong basis for developing individualized control strategies based on genotype in the clinical management of MAC-PD in the future. These suggestions may contribute to reducing the unnecessary use of antibiotics by reducing relapses and ultimately improving long-term outcomes for affected patients through a combination of molecular microbiology, clinical care, and public health surveillance.

## CONCLUSIONS

Management of MAC-PD remains a clinical challenge due to its high rate of recurrence and the inherent difficulty in distinguishing relapse from reinfection. Strain-level genotyping has emerged as a key approach to resolve this diagnostic uncertainty, enabling more informed treatment decisions and improved outcomes. A growing body of evidence supports the utility of high-resolution tools, particularly WGS and MLST, for accurately classifying recurrence mechanisms based on genomic variation. These approaches are complemented by pattern-based methods such as rep-PCR and MIRU-VNTR, which offer practical advantages in speed and accessibility, especially in routine clinical contexts. Beyond the diagnosis of recurrence, genotyping data offer critical value in the personalization of antimicrobial strategies, environmental source tracking, and infection control planning. To realize these benefits more broadly, several practical strategies are warranted: routine paired-isolate genotyping, multi-colony analysis, harmonized SNP-based interpretive thresholds, the establishment of a global MAC strain database, and the integration of genotypic data into clinical decision-support systems. Additionally, emerging technologies such as CRISPR-based strain typing may present new opportunities for rapid, culture-independent strain discrimination.

Together, these tools and strategies can help close the gap between molecular diagnostics and real-world decision-making in MAC-PD. Their integration into routine care has the potential to reduce misclassification, inform more targeted interventions, and ultimately contribute to better clinical outcomes and stronger public health responses in the face of this increasingly prevalent and complex disease.
